# Supplementary material for: The origins and relatedness structure of mixed infections vary with local prevalence of P. falciparum malaria
Source: eLife. 2019 Jul 12;8:e40845. doi: 10.7554/eLife.40845 (PMC6684230; doi:10.7554/eLife.40845)
Supplement: Supplementary file 1. [file elife-40845-supp1.pdf]

About the Pf3k Project

The analyses in this paper are based on the Pf3k version 5 dataset, released in February 2016, comprising Illumina short read sequence data and SNP/indel calls on 2,512 field samples, 5 cultured parasite lines, 96 samples from genetic crosses, and 27 samples from artificial mixtures of cultured parasite lines.

Background

The Pf3k Project was established in 2014 to develop high quality open access data resources for analysis of *Plasmodium falciparum* genome variation, based on standardised protocols for variant discovery and genotype calling, together with new tools for analysis of complex genetic variation in field samples (https://www.malariagen.net/projects/pf3k).

Field samples from Bangladesh, Cambodia, Democratic Republic of Congo, Guinea, Laos, Malawi, Mali, Myanmar, Nigeria, Thailand and The Gambia were sequenced as part of the MalariaGEN *Plasmodium falciparum* Community Project (https://www.malariagen.net/projects/p-falciparum-community-project). As these samples were collected by different MalariaGEN partner studies over a period of several years, and the sequence data have been made openly available, previous analyses of subsets of these samples can be found in publications by the MalariaGEN network and its partner studies (refs 1-12). Further details of individual partner studies are given below.

All sequencing data were produced by the Wellcome Sanger Institute (refs 1-12,15,16), with the exception of 137 samples from Senegal that were sequenced by the Broad Institute (ref 13,14). The sequence data on parents and progeny of genetic crosses have been previously published by Miles et al, 2016 (ref 15). The artificial mixtures of cultured parasite lines were contributed by Jason Wendler and Kirk Rockett at the Wellcome Trust Centre for Human Genetics and the Wellcome Sanger Institute. In another part of the Pf3k Project, PacBio long read sequence data were produced on 15 cultured isolates from different geographical locations, as reported by Otto et al, 2018 (ref 16).

Pf3k Project data can be openly accessed at https://www.malariagen.net/data and can be browsed using the interactive Panoptes web application (ref 17) at https://www.malariagen.net/apps/pf3k/ .

Members of the Pf3k Project

Steering group: Matt Berriman, Dominic Kwiatkowski, Gil McVean, Dan Neafsey

Population genetic working group: Jacob Almagro-Garcia, Roberto Amato, Zam Iqbal, Dominic Kwiatkowski, Sorina Maciuca, Gil McVean, John O'Brien, Joe Zhu.

Technology benchmarking working group: Roberto Amato, Dominic Kwiatkowski, Dan Neafsey, Richard Pearson, Jim Stalker

Reference genome working group: Matt Berriman, Chris Newbold, Thomas Otto

Coordination: Vikki Cornelius, Bronwyn MacInnis, Dawn Muddyman

Partner study lead investigators: Alfred Amambua-Ngwa, Lucas Amenga-Etego, Gordon Awandare, David Conway, Alistair Craig, Nicholas Day, Abdoulaye Djimde, Arjen Dondorp, Rick Fairhurst, Olivo Miotto, Nick White

MalariaGEN partner studies contributing to the Pf3k Project

Study 1001: Developing the Community Project with partners in Mali

- Description: https://www.malariagen.net/network/where-we-work/1001-developing-community-project-partners-mali
- Key contact: Abdoulaye Djimdé (adjimde@icermali.org)

Study 1006: Genome-wide analysis of genetic variation in The Gambia

- Description: https://www.malariagen.net/network/where-we-work/1006-genome-wide-analysis-genetic-variation-gambia
- Key contact: Alfred Amambua-Ngwa (angwa@mrc.gm)

Study 1017: Population genetics of natural populations in Northern Ghana

- Description: https://www.malariagen.net/network/where-we-work/1094-population-genetics-p-falciparum-parasites-northern-ghana
- Key contact: Lucas Amenga-Etego (lucasmenga@gmail.com)

Study 1022: Genome variation and selection in clinical isolates from rural Malawi

- Description: https://www.malariagen.net/network/where-we-work/1022-genome-variation-and-selection-clinical-isolates-rural-malawi
- Key contact: Alister Craig (alister.craig@lstmed.ac.uk)

Study 1026: Effects of transmission intensity on population structure and signatures of selection in Guinea

- Description: https://www.malariagen.net/network/where-we-work/1026-effects-transmission-intensity-population-structure-and-signatures
- Key contact: David Conway (David.Conway@lshtm.ac.uk)

Study 1044: Genomics of Plasmodium clearance and recrudescence rates in Cambodia

- Description: https://www.malariagen.net/network/where-we-work/1044-genomics-plasmodium-clearance-and-recrudescence-rates-cambodia
- Key contact: Thomas E. Wellems (twellems@niaid.nih.gov)

Study 1052: Tracking Resistance to Artemisinin Collaboration (TRAC)

- Description: https://www.malariagen.net/network/where-we-work/1052-tracking-resistance-artemisinin-collaboration-trac
- Key contact: Elizabeth Ashley (liz@tropmedres.ac.uk)

Study 1083: Alternative molecular mechanisms for erythrocyte invasion by P. falciparum in Ghana

- Description: https://www.malariagen.net/network/where-we-work/1083-alternative-molecular-mechanisms-erythrocyte-invasion-p-falciparum-ghana
- Key contact: Gordon Awandare (gawandare@ug.edu.gh)

Study 1094: Population genetics of P. falciparum parasites in Northern Ghana

- Description: https://www.malariagen.net/network/where-we-work/1094-population-genetics-p-falciparum-parasites-northern-ghana
- Key contact: Lucas Amenga-Etego (lucasmenga@gmail.com)

References

1. Auburn et al (2011). An effective method to purify Plasmodium falciparum DNA directly from clinical blood samples for whole genome high-throughput sequencing. PLoS One 6, e22213. DOI:10.1371/journal.pone.0022213
2. Auburn et al (2012). Characterization of within-host Plasmodium falciparum diversity using next-generation sequence data. PLoS One 7, e32891. DOI:10.1371/journal.pone.0032891
3. Amambua-Ngwa et al (2012). Population genomic scan for candidate signatures of balancing selection to guide antigen characterization in malaria parasites. PLoS Genetics 8, e1002992. DOI: 10.1371/journal.pgen.1002992
4. Manske et al (2012). Analysis of Plasmodium falciparum diversity in natural infections by deep sequencing. Nature 487, 375-9. DOI: 10.1038/nature11174
5. Ashley et al (2014). Spread of artemisinin resistance in Plasmodium falciparum malaria. Tracking Resistance to Artemisinin Collaboration (TRAC). N Engl J Med. 371, 411-23. DOI: 10.1056/NEJMoa1314981.
6. Miotto et al (2013). Multiple populations of artemisinin-resistant *Plasmodium falciparum* in Cambodia. Nature Genetics 45, 648-55. DOI: 10.1038/ng.2624
7. Miotto et al (2015). Genetic architecture of artemisinin-resistant Plasmodium falciparum. Miotto et al. Nature Genetics 47, 226-34. DOI: 10.1038/ng.3189
8. Kamua et al (2015). K13-propeller polymorphisms in Plasmodium falciparum parasites from sub-Saharan Africa. Journal of Infectious Diseases 211, 1352-5 DOI: 10.1093/infdis/jiu608
9. MalariaGEN *Plasmodium falciparum* Community Project (2016) Genomic epidemiology of artemisinin resistant malaria. eLife 5, e08714. DOI: 10.7554/eLife.08714
10. Duffy et al (2015). Comparison of genomic signatures of selection on *Plasmodium falciparum* between different regions of a country with high malaria endemicity. BMC Genomics 16, 527 DOI: 10.1186/s12864-015-1746-3
11. Amato et al (2016). Genetic markers associated with dihydroartemisinin–piperaquine failure in Plasmodium falciparum malaria in Cambodia: a genotype–phenotype association study. Lancet Infectious Diseases, DOI: 10.1016/S1473-3099(16)30409-1
12. Amato et al (2018). Origins of the current outbreak of multidrug-resistant malaria in southeast Asia: a retrospective genetic study. Lancet Infect Dis. 18, 337-345. doi: 10.1016/S1473-3099(18)30068-9.
13. Chang et al (2012). Genomic sequencing of Plasmodium falciparum malaria parasites from Senegal reveals the demographic history of the population. Mol Biol Evol. 29, 3427-39. doi: 10.1093/molbev/mss161
14. Chang et al (2013). Malaria life cycle intensifies both natural selection and random genetic drift. Proc Natl Acad Sci U S A. 110, 20129-34. doi: 10.1073/pnas.1319857110
15. Miles et al (2016). Indels, structural variation, and recombination drive genomic diversity in Plasmodium falciparum. Genome Res. 26, 1288-99. doi: 10.1101/gr.203711.115
16. Otto et al (2018). Long read assemblies of geographically dispersed Plasmodium falciparum isolates reveal highly structured subtelomeres. Wellcome Open Res. 3:52. doi: 10.12688/wellcomeopenres.14571.1.
17. Vauterin et al (2017). Panoptes: web-based exploration of large scale genome variation data. Bioinformatics 33, 3243-3249. doi: 10.1093/bioinformatics/btx410.
